# Spatial homogeneity pursuit of regression coefficients for hand, foot and mouth disease in Xinjiang Uygur Autonomous Region in 2018

**DOI:** 10.1038/s41598-022-26003-6

**Published:** 2022-12-12

**Authors:** Xiaoshuang Zhong, Peilin Wang, Huiguo Zhang

**Affiliations:** College of Mathematics and System Science, Xinjiang University, Urumqi, 830017 China

**Keywords:** Public health, Statistics, Diseases, Infectious diseases

## Abstract

To explore the complex spatial pattern between the incidence of hand, foot, and mouth disease (HFMD) and meteorological factors [average temperature (AT), average relative humidity (ARH), average air pressure (AP), average wind speed (AW)], this paper constructed a Spatial Clustering coefficient (SCC) regression model to detect spatial clustering patterns of each regression coefficients in different seasons. The results revealed that compared with geographically weighted regression (GWR), the coefficients estimated by SCC method were more smooth with clearly identified spatial and improved edge effects. Therefore, interesting spatial patterns were easy to identify in the SCC estimated coefficients. And then, the SCC method had better estimation accuracy in estimating the relationship between potential meteorological factors and HFMD cases. Meteorological factors had different significance in their effect on HFMD incidence depending on the season. Specifically, the influence of AT on HFMD was negatively correlated in summer and winter, especially in the Altay region, Bayingoleng Mongolian Autonomous Prefecture, Turpan region and Hami region. Second, AW had positive effects with HFMD in summer, but the AW played a negative role in the whole Xinjiang in winter. In Tianshan district, Shayibake district, Shuimogou district, etc. in summer, ARH showed a strong negative correlation, but in Alar city it had a high positive correlation, however, in winter ARH showed a high negative correlation in Altay regions, Aksu region and other places had negative effects, and it showed a strong positive correlation in Shayibak district. Finally, AP had a strong positive correlation with HFMD in summer in Shaybak district, but in winter, AP showed a strong negative correlation in Altay district and Buxel Mongolia Autonomous county. In summary, Xinjiang should adapt measures to local conditions, and formulate appropriate HFMD prevention strategies according to the characteristics of different regions, time, and meteorological factors.

## Introduction

Hand, Foot and Mouth Disease (HFMD) is a viral(enterovirus), infectious and global disease that occurs frequently in children under the age of 5. The Ministry of Health of China designated HFMD as a ”C” infectious disease in May 2008. In recent years, it has occurred all over China throughout the year. The threat of HFMD to public health has promoted scholars to study the characteristics and causes of the disease. Many researchers focused on the impact of meteorological factors on HFMD. Studies have shown that meteorological factors such as average temperature and average relative humidity will affect the incidence of HFMD^1–5^. Ma et al. found that the HFMD visit rate in Hong Kong was positively correlated with the average temperature, daily temperature difference, relative humidity, and wind speed^6^. And in Guangzhou and Shenzhen, by time series analysis, they revealed that temperature was positively correlated with the incidence of HFMD^7,8^. But a Japanese study found that the number of days per week with an average temperature exceeding 25 °C was negatively correlated with the incidence of HFMD^9^. However, the above studies did not consider the spatial differences in the incidence of HFMD. The GWR model, as a spatial statistics method can effectively to model and analyze the heterogeneity of space. Hu et al. used GWR model to investigate the potential factors affecting the incidence of HFMD in children^10^. Similarly, Hong et al. used GWR model to discuss the relationship between HFMD and meteorological factors in Inner Mongolia, and they discovered that there was a correlation between these factors and HFMD, and each factor had its unique spatial heterogeneity^11,12^, as demonstrated by Wang et al. and Bo et al.^13^.

It is common knowledge that GWR models tend to estimate regression coefficients as continuous functions, however for data collected from a large area, the relationship between response variables and predictors may exhibit complex spatial dynamic patterns, and the variation of regression coefficients is not necessarily continuous. In particular, relationships between spatial variables may change abruptly at the boundaries of adjacent clusters but remain relatively homogeneous within clusters^14–16^. Fortunately, the study on the homogeneity pursuit of regression coefficients in high-dimensional data analysis will help to solve this problem. In these studies^17,18^, pairwise coefficient differences are penalized to encourage homogeneity among coefficients. According to these ideas, Li et al. proposed Spatial Clustering Coefficient (SCC) regression to detect the spatial clustering pattern of regression coefficients, which integrates spatial domain information by constructing appropriate regularization to automatically detect mutation points in regression coefficients, and due to its relatively strong local adaptability, it can also estimate continuously changing regression coefficients^19^. Therefore, this paper was to detect and analyze the spatial clustering patterns of AT, MT, ARH, AP, and AW on HFMD occurrence in Xinjiang in 2018 using the SCC model at county-level and monthly-scale.

## Results

### Statistical analysis

**Figure 1:**
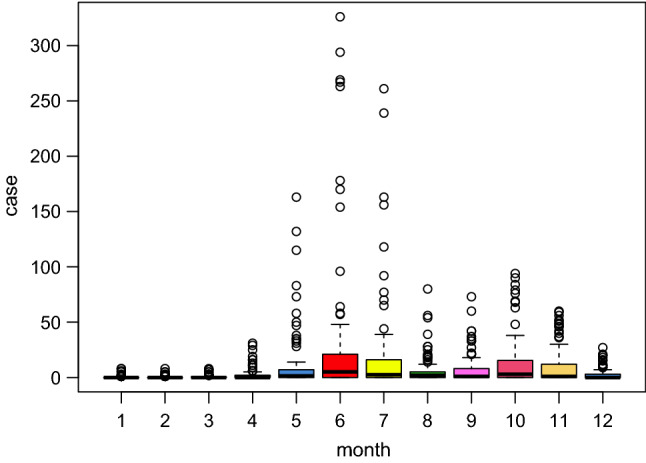
The monthly distribution of HFMD cases in Xinjiang, China, 2018.

From January 1 to December 31, 2018, the total number of HFMD cases in 100 counties in Xinjiang was 10,260. The monthly distribution of HFMD cases in Xinjiang is shown in Fig. 1. The peak of HFMD occurred in May–July, and the second peak occurred in September–November. Therefore, May–December is chosen as the main research period.

**Figure 2:**
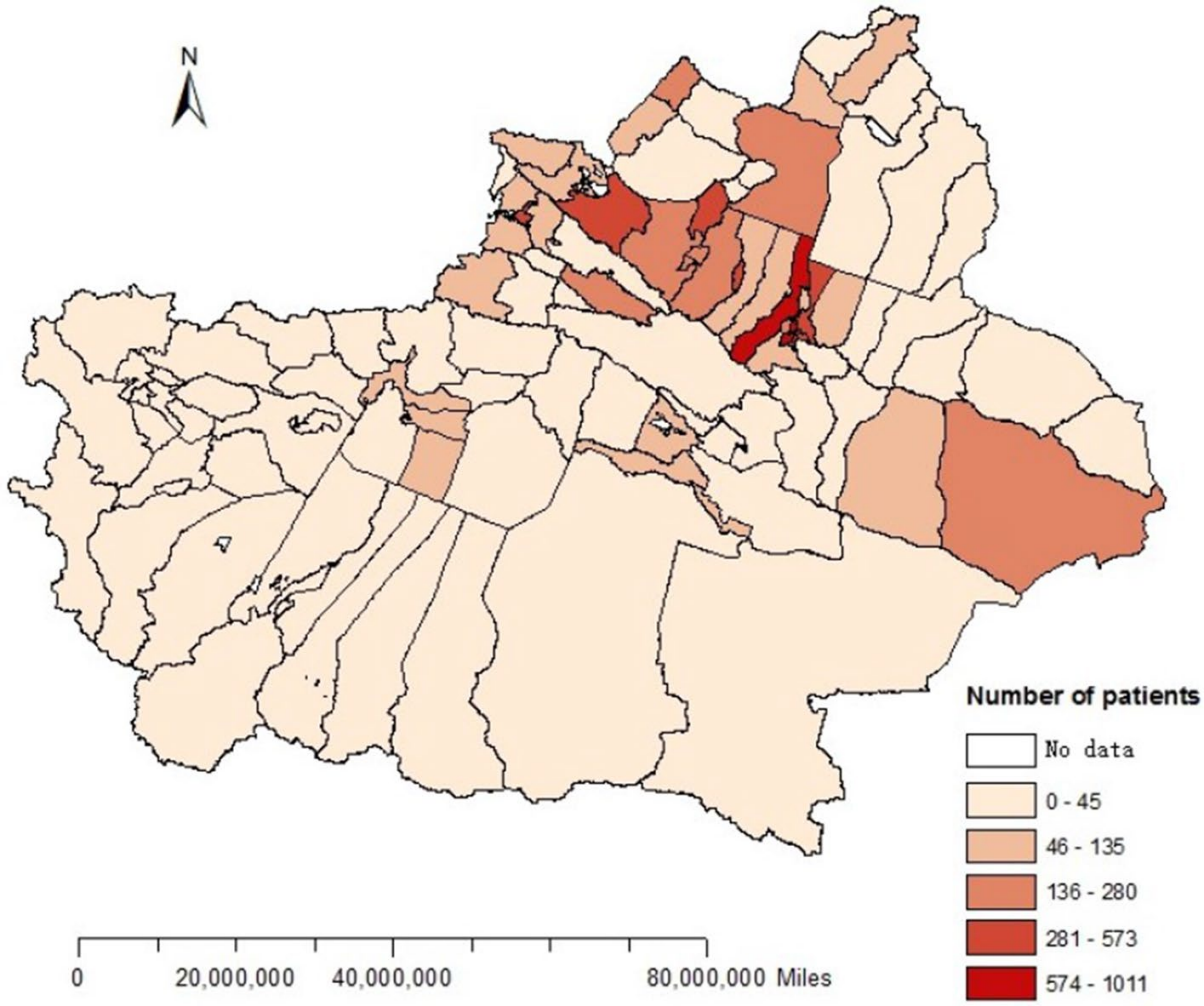
Distribution characteristics of HFMD cases from May to December in Xinjiang.

The distribution of HFMD cases in Xinjiang from May 1 to December 31, 2018 is shown in Fig. 2. The results revealed that the incidence of HFMD in northern Xinjiang is higher than that in southern Xinjiang, and the high incidence areas include Changji city, Xinshi district, Shayibak district, Shuimogou district, Midong district, Tianshan district, Shihezi city, Yining city, Jinghe county, Karamay district and Yizhou district.

**Figure 3:**
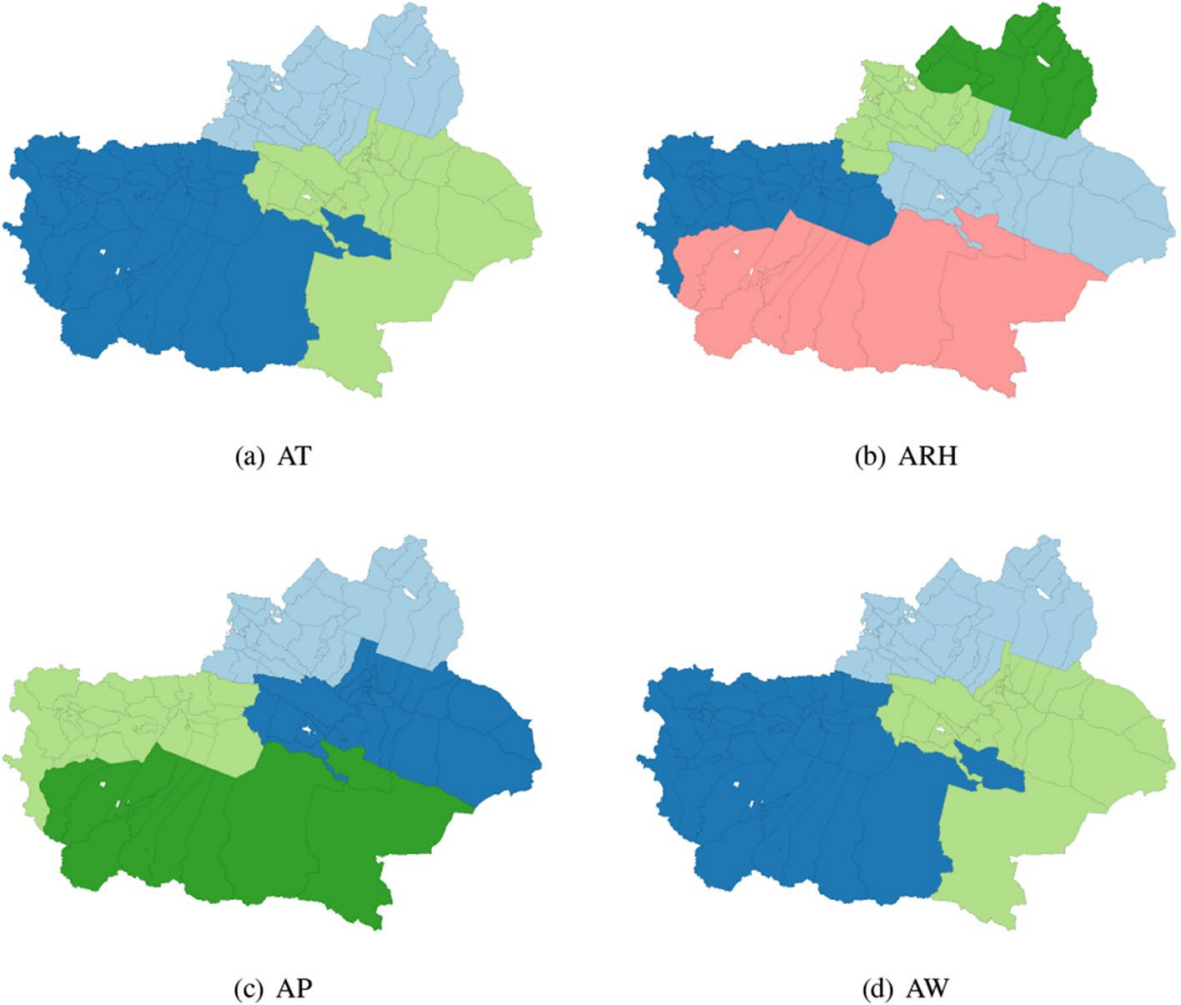
Spatial cluster distribution of some meteorological factors (AT, ARH, AP, AW) in Xinjiang in June 2018.

It can be clearly seen from Fig. 3 that the temperature distribution in Altay region and Tacheng region is similar, and the temperature distribution in Ili Kazakh Autonomous Prefecture, Aksu, Kizilsu Kirgiz Autonomous Prefecture, Kashi region and Hotan region are the same. For ARH, Altay region is a category, Ili Kazakh Autonomous Prefecture is a category, Aksu region and Kizilsu Kirgiz Autonomous Prefecture are clustered into a category, and Kashi region, Hotan region and part of Bayingoleng Mongolian Autonomous Prefecture are a category. The spatial clustering of AP is similar to that of ARH, and AW distribution is similar to that of AT. The specific spatial clustering results are shown in Fig. 3.

### Correlation analysis of meteorological factors

Spearman correlation coefficient analysis is used to explore the relationship between the incidence of HFMD (NUM) and meteorological factors in Xinjiang. The detailed results are shown in Table 1. The results reveal that the correlation between AT and MT is 0.968, which is highly correlated. Therefore, meteorological factors other than MT are selected as explanatory variables when establishing the SCC model.

**Table 1:** Correlation analysis of NUM, AT, MT, ARH, AP, and AW, where the values represent correlation, and ***means that p-value is less than 0.01, that is, there is significant correlation between variables.

	NUM	AT	MT	ARH	AP	AW
NUM	1.000					
AT	− 0.439***	1.000				
MT	− 0.486***	0.950	1.000			
ARH	0.511***	− 0.665	− 0.632	1.000		
AP	0.597***	− 0.271	− 0.283	0.480	1.000	
AW	0.064	− 0.354	− 0.376	− 0.127	0.099	1.000

**Table 2:** The SCC results of the regression coefficients in June, where NSCC means number of spatial clustering categories.

	NSCC	Spatial Clustering Area
AT	3	I: Bayingoleng Mongolian Autonomous Prefecture,Turpan region, Hami region
		II: Altay region, and Busier Mongolia Autonomous County, Wuerhe district, Baijiantan district, Karamay district
		III: The northern part of Tacheng area,Bortala Mongolian Autonomous Prefecture, Ili Kazakh Autonomous Prefecture, Aksu Prefecture, Kizilsu Kirgiz Autonomous Prefecture, Kashgar Prefecture, Hotan Prefecture
ARH	5	I: Tianshan district, Shayibak district,Shuimogou district, Midong district, Wujiaqu city, Fukang city
		II: Hami area, Mulei Kazakh Autonomous county, Qitai county, Jimsar county
		III: Remaining areas except I,II,IV,V
		IV: Altay region, Tacheng region, Manas county, Shihezi city, Hutubi county, Changji Hui Autonomous Prefecture
		V: Aral city
AP	5	I: Aksu city
		II: Kashgar city, Artush city, Aheqi county, Keping county, Wushi county, Wensu county, Awati county, Alar city
		III: Altay region (except Jimunai county), Fukang city, Jimsar county, Qitai county
		IV: Remaining areas except I,II,III,V
		V: Xinshi district, Sayibak district, Tianshan district, Shuimogou district
AW	3	I: Altay region,Tacheng region, Changji Hui Autonomous Prefecture, Hami region, Turpan region, Bayingoleng Mongolian Autonomous Prefecture
		II: Remaining areas except I,III
		III: Aksu region, Arche county, Wushi county, Keping county, Bachu county, Tumushuke city, Awati county, Wensu county

### Spatial cluster analysis of regression coefficient

**Figure 4:**
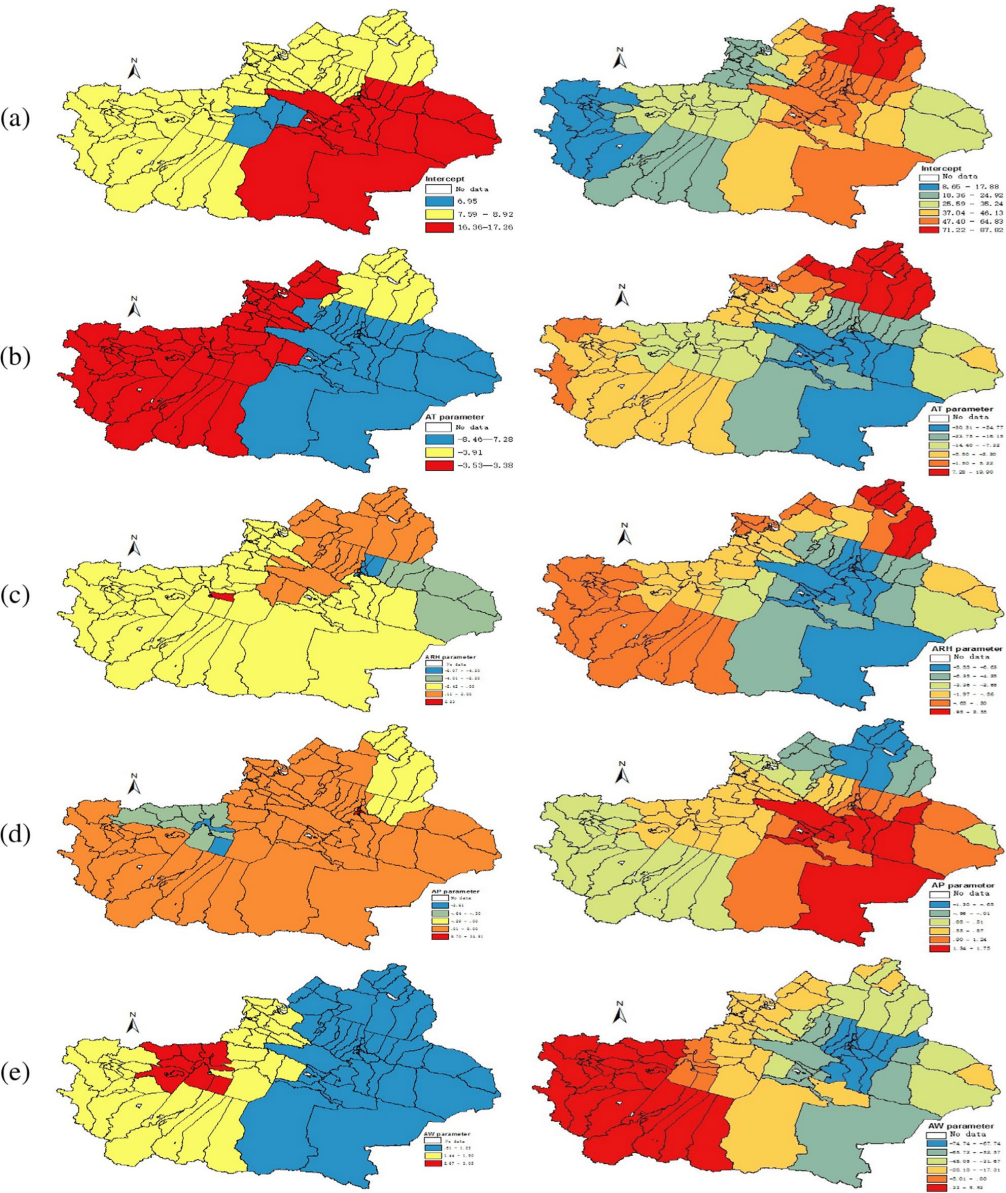
Spatial variation surface of SCC (left panel) and GWR (right panel) model estimation parameters in June 2018, and the estimated regression coefficients of Intercept, AT, ARH, AP, AW in rows (**a**–**e**), respectively.

**Figure 5:**
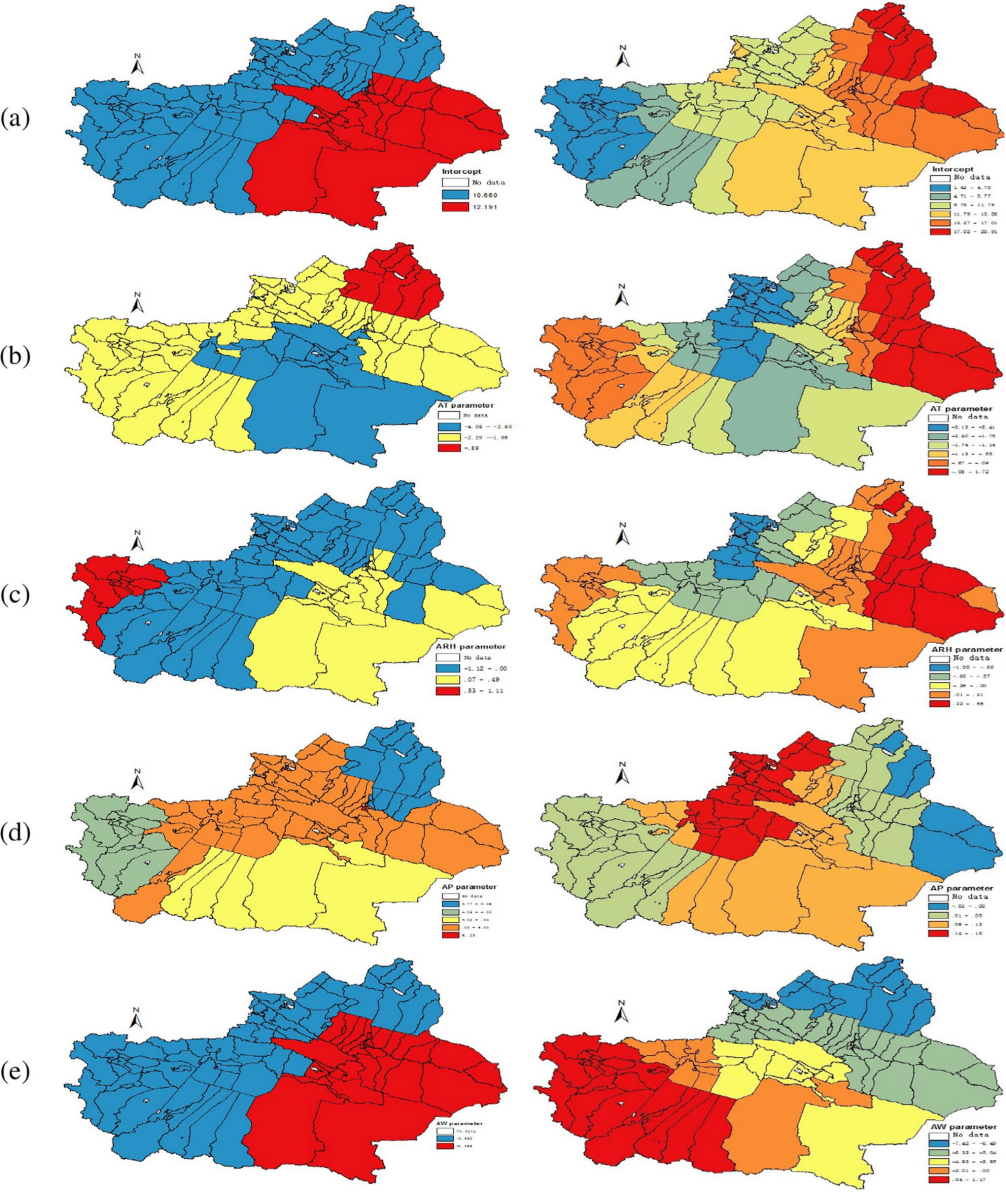
Spatial variation surface of SCC (left panel) and GWR (right panel) model estimation parameters in Nov. 2018, and the estimated regression coefficients of Intercept, AT, ARH, AP, AW in rows (**a**–**e**), respectively.

In order to compare the spatial clustering patterns of the regression coefficients in different time periods, this paper mainly selects the data of June and November for analysis, because the statistical analysis shows that the number of HFMD cases in June and November is higher. Next, the partial coefficient estimates obtained by the SCC method are shown in Figs. 4 and 5. At the same time, this paper also shows the coefficient estimates obtained by the GWR method, which is convenient for comparison and verification.

By comparing Figs. 4 and 5 , it can be seen that the estimated coefficients ${\varvec{{\beta }}}{} \mathbf{(u,v)}$ vary spatially throughout different seasons. First of all, the incidence of HFMD in southeastern Xinjiang is higher than that in other regions in June 2018. (the first map of SCC in Figs. 4 and 5). Secondly, the AT in June has a negative correlation with HFMD, and the impact categories are mainly divided into three categories, among which it plays an important role in Bayingoleng Mongolian Autonomous Prefecture, Turpan region, Hami region, Changji, Shihezi and Urumqi, in the Altay region and Hebusaier Mongolian Autonomous Prefecture, Wuerhe county, Baijiantan district and Karamay district, the impact is moderate and in the northern part of Tacheng region, Bortala Mongolian Autonomous Prefecture, Ili Kazakh Autonomous Prefecture, Aksu Region, Kizil Sukyrgiz Autonomous Prefecture, Kashgar and Hotan have weaker effects. The ARH in Tianshan district, Shayibak district, Shuimogou district, Midong district, Wujiaqu city and Fukang county is highly negatively correlated with HFMD cases, but the ARH is strongly positively correlated with the incidence of HFMD in Aral city. The AT has a positive correlation in most areas of Xinjiang, with strong explanatory power in Xinshi district, Shayibak district, Tianshan district and Shuimogou district, but it has a negative correlation in Aksu, which is mainly divided into two levels, among which Aksu shows a high negative correlation, and the influence in other areas is small. The AW shows a positive correlation in the whole Xinjiang, which is roughly divided into three levels of positive correlation, especially in Aksu and some parts of Kashgar, it is strongly correlated. To show the clustering results more clearly, the spatial clustering patterns of these regression coefficients are summarized in Table 2.

In November 2018, the HFMD cases in Xinjiang is mainly distributed according to two characteristics. Specifically, the incidence of HFMD in Bayingoleng Mongolia Autonomous Prefecture, Turpan, Hami and other regions was 12.191 and HFMD cases in other areas of Xinjiang is 10.66. AT shows a negative impact, among which the central and southeast Xinjiang shows a strong effect, and the influence intensity in the Altay region is constant − 0.89. The ARH shows a strong positive correlation in parts of Kyzylsu Kirgiz Autonomous Prefecture, but in the Altay region, the Tacheng region, Borta Lameng Autonomous Prefecture, Ili Kazakh Autonomous Prefecture, Kashgar region of Aksu region, and Hotan region, it shows a strong negative correlation. The AP shows a strong negative correlation in the Altay region, but a high positive correlation in the Saybak region. The AW shows a negative correlation in the whole Xinjiang, which is roughly divided into two levels of positive correlation.

Compared with GWR, the coefficient estimated by SCC has a clearer clustering effect and clear spatial pattern recognition. The reason maybe is the coefficients estimated by GWR are rough and noisy in the study area. Therefore, in the coefficients estimated by GWR, the spatial patterns of clusters are difficult to identify. And by observing Figs. 3 and 4, it is found that the spatial clustering pattern of the regression coefficients is highly similar to the spatial distribution characteristics of meteorological factors, which indicates that the SCC method can accurately identify the real spatial characteristics of the regression coefficients studied in this paper.

### The SCC Model evaluation and analysis

In order to intuitively judge the superiority of SCC, this paper calculates the coefficient of determination of the regression model based on the data of June and November, the estimated number of HFMD cases and the mean square error (MSE) of the number of cases per month.

**Table 3:** The coefficient of determination under GWR estimation and SCC estimation.

	June	November
SCC	0.999	0.999
GWR	0.383	0.271

**Figure 6:**
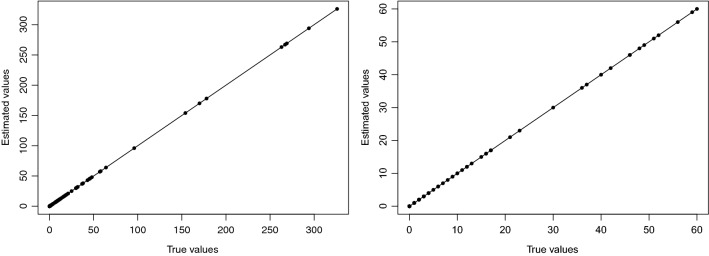
The curve of HFMD incidence estimated based on SCC model and actual incidence, and left panel is in June, 2018 and the right panel is in November, 2018.

**Figure 7:**
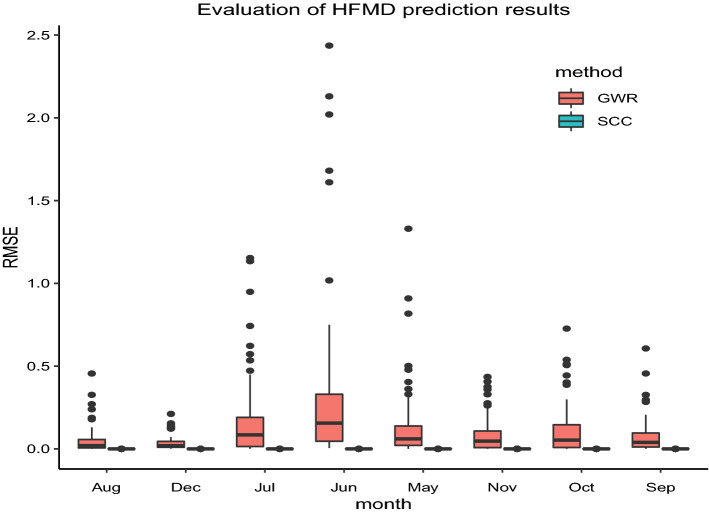
RMSE comparison results based on SCC and GWR.

From the observation of Fig. 6 and Table 3, it can be seen that the curve drawn by the actual patient value and the estimated value based on SCC model presents a 45 straight line, and the coefficient of determination estimated based on the SCC method is close to 1, indicating that the estimated value and the true value are almost the same, thus illustrating the accuracy of the SCC model estimation. To further illustrate the accuracy of SCC model estimation, this paper compares SCC and GWR model estimation results, and the results are shown in Fig. 7. The MSE values estimated by SCC are all around 0 and better than the estimated by GWR.

## Discussion

Spatial epidemiology can clearly understand the spatial clustering of diseases, explore the clustering location and exact scope through spatial analysis models^20^. At present, many studies have carried out spatial correlation analysis on the characteristics of the number of people infected with various infectious diseases, which have not been considered. The influence of other factors on the number of infectious diseases^21–23^. Moreover, there are few spatial analyses of the incidence of HFMD in Xinjiang, and relevant studies have focused on the analysis of epidemiological signs^24^.

In this study, based on the dataset of 100 counties and cities in Xinjiang, Spatial Clustering Coefficient (SCC) regression is used to detect the spatial clustering patterns of regression coefficient and estimates the regression coefficient values, so as to speculate the influence pattern of meteorological factors on HFMD cases. The results show that the regression coefficients have different spatial clustering patterns for different seasons. Compared with previous studies^25–27^, the AT, ARH, AP and AW factors all have an impact on the number of HFMD cases in Xinjiang. The existing studies have shown that the incidence of HFMD in northern Xinjiang is higher because of its high population density, which is consistent with the results of this study. Secondly, the study finds HFMD cases decline as AT rise in June. Some studies have been shown that the influence of temperature on HFMD is inverted “U”. When the temperature is lower than a certain value, the incidence of HFMD increases with the increase of temperature, whereas the risk of HFMD decreases with the increase of temperature^4,7^. And Xinjiang is in the midsummer season in June, because the daytime average temperature is as high as 28 °C, which leads to a decrease in the number of people going out, thus reducing the incidence of HFMD^1^. On the contrary, AW has a positive impact on the number of HFMD cases. It may be that the increased wind speed promotes the spread of the virus and the higher wind speed will cause cool weather, which will further promote people’s outdoor activities, so the incidence of HFMD increases. The regression coefficients of ARH have five spatial clustering patterns. First, with the increase of ARH, the incidence of HFMD decreases fastest in Urumqi and its surroundings compared with other areas, and at the same time in Aral City, with the increase of ARH, the incidence of HFMD increased rapidly. AP plays an important role in Xinshi District, Sayibak District, and Tianshan District, while it shows a strong negative correlation in Aksu District, and a study in Guangdong revealed that for every 1 hPa increase in air pressure, the number of cases decreased by 6.8^28^. This may be because lower air pressure may weakens the human’s/organism’s immune system^29^. In early winter (November), Similar to June, AT has a negative correlation with the incidence of HFMD and its regression coefficients have three cluster, but its influence intensity is relatively weak, and it has a strong explanatory power in Bayingoleng Autonomous Prefecture and other areas. The reason may be that as the temperature dropped, the virus began to multiply in large numbers, and susceptible people gathered, which accelerated the spread of the virus^28^. The above results fully reflect the seasonal characteristics of enterovirus^6,9^. AW and HFMD cases shows a negative correlation in Xinjiang. According to a systematic review on the association between ventilation and infection suggested that higher ventilation rates could decrease infection rates or outbreaks of some airborne diseases^30^. Hence, it is likely that strong natural ventilation could serve as a barrier for the spread of respiratory droplets. For the regression coefficients of ARH, it has relatively complex spatial patterns, and it may seem that the ARH in each region has a different impact on the number of HFMD cases due to the complex terrain of Xinjiang. According to previous research, in days with high relative humidity, the infected people may excrete more enteroviruses into the environment^31^, and these enteroviruses can easily attach to the surface of toys or small particles in the air, and the process may cause the virus to accumulate in the environment, thereby accelerating the spread of HFMD^32^. This is consistent with the results of this study in November (except for the northern, northwestern, and southwestern regions of Xinjiang).

In summary, the coefficient spatial clustering patterns of AT, ARH, AP and AW in June and November in Xinjiang are detected by using the SCC method, and the influence characteristics of meteorological factors on HFMD are analyzed. It is hoped that this will provide some theoretical basis for prevention and control of HFMD in Xinjiang.

## Methods

### Data source

The research data of this paper are the number of confirmed HFMD cases in 100 cities and counties (municipal districts) in Xinjiang from January 2018 to December 2018, and the daily incidence of HFMD in Urumqi, Xinjiang in 2018, as well as five meteorological factors: daily average temperature (°C), minimum temperature (°C), average relative humidity (%), average air pressure (Pa) and average wind speed (m/s). The data are from the Public Health Science Data Center of Chinese Center for Disease Control and Prevention (http://www.phsciencedata.cn/Share/index.jsp).

### Model

Suppose the spatial data $(x(u_{i},v_{i}),y(u_{i},v_{i})),i = 1,\cdots ,n$ is the observation of the observation position $(u_{1},v_{1}),\cdots ,(u_{n},v_{n})\in R^{2}$, where the response variable $y(u_{i},v_{i})$ is spatially correlated and ${\textbf{x}}(u_{i},v_{i})=(x_{1}( u_{i},v_{i}),\cdots ,x_{p}(u_{i},v_{i}))^{'}$ is p dimensional explanatory variables at $(u_{i},v_{i})$. Consider a spatial variable coefficient model

1 $$\begin{aligned} y(u_{i},v_{i})=\sum \limits _{k =1}^{p}x_{k}(u_{i},v_{i})\beta _{k}(u_{i},v_{i})+\epsilon (u_{i},v_{i}). \end{aligned}$$

Clearly, this problem needs to be regularized because there are more variables and parameter than observations. For spatial problems, the association between the response variable and the explanatory variables at nearby locations is expected to be highly homogenous, which prompted us to assign $\varvec{\beta }$ a regularization function that reflects this spatial homogeneity.

Specifically, the paper minimizes the SCC model to estimate ${\varvec{{\beta }}}$

2 $$\begin{aligned} \frac{1}{n}\sum \limits _{i = 1}^{n}[y(u_{i},v_{i})-\sum \limits _{k = 1}^{p}x_{k}(u_{i},v_{i})\beta _{k}(u_{i},v_{i})]^{2} + \lambda \sum \limits _{k = 1}^{p}\sum \limits _{(i,j)\in E}|\beta _{k}(u_{i},v_{i})-\beta _{k}(u_{j},v_{j})|. \end{aligned}$$

where E is an edge set consisting of n vertices, each of which corresponds to an observation position, and $|\cdot |$ is a generalized lasso penalty function to judge the corresponding positions of the two regression coefficients $(u_{i},v_{i})$ and $(u_{j},v_{j})$ are connected by an edge in E, thus encouraging homogeneity between the two regression coefficients. $\lambda$ is a penalty parameter, which is selected using the BIC criterion in this example. Once an edge set E is given, (2) is written in matrix form

3 $$\begin{aligned} \frac{1}{n}\sum \limits _{i = 1}^{n}[y(u_{i},v_{i})-\sum \limits _{k = 1}^{p}x_{k}(u_{i},v_{i})\beta _{k}( u_{i},v_{i})]^{2}+\lambda \sum \limits _{k = 1}^{p}|{\textbf{H}}{\varvec{{\beta }}}_{k}|_{1}. \end{aligned}$$

where ${\textbf{H}}$ is an $m\times n$ matrix consisting of an edge set E with m edges. For the edge connecting two positions $(u_{i},v_{i})$ and $(u_{j},v_{j})$ this paper denote the penalty term $| \beta _{k}(u_{i},v_{i})-\beta _{k}(u_{j},v_{j})|$ as $|{\textbf{H}}_{m}{\varvec{{\beta }}}_{k}|$, ${\textbf{H}}_{m}$ represents the row vector of ${\textbf{H}}$, which contains only two non-zero elements, the i-th element is 1, and the j-th element is − 1.

The marginal set E is a key component of the SCC model because it reflects prior assumptions about the structure of the regression coefficients. This paper constructs the edge set E by generating a minimum spanning tree (MST).

Once the MST is constructed, the resulting penalty no longer contains redundant terms, so the original problem can be easily transformed into a lasso or lasso-type problem after appropriate reparameterization. Define new parameters ${\varvec{{\theta }}}_{k},k = 1,\cdots ,p$ as $${\varvec{{\theta }}}_{k} = \begin{pmatrix} {\textbf{H}} \\ \frac{1}{n}{\textbf{1}}^{T} \\ \end{pmatrix}{\varvec{{\beta }}}_{k} = \widetilde{{\textbf{H}}}{\varvec{{\beta }}}_{k}$$.

This new design matrix is written as $\widetilde{{\textbf{X}}} =[diag({\textbf{X}}_{1})\widetilde{{\textbf{H}}}^{- 1},\cdots ,diag({\textbf{X}}_{n})\widetilde{{\textbf{H}}}^{- 1}]$, where $\widetilde{{\textbf{H}}}$ is an $n\times n$ invertible matrix, Since ${\textbf{H}}$ rows are full rank, there is a one-to-one transformation between ${\varvec{{\beta }}}_{k}$ and ${\varvec{{\theta }}}_{k}$. Then, the SCC model in equation 4 can be rewritten as

4 $$\begin{aligned} \frac{1}{n}|{\textbf{y}} -\widetilde{{\textbf{X}}}{\varvec{{\theta }}}|_{2}^{2}+\lambda \sum \limits _{l \in B}| \theta _{l}|. \end{aligned}$$

where ${\varvec{{\theta }}}=({\varvec{{\theta }}}_{1}^{'},\cdots ,{\varvec{{\theta }}}_{p}^{'})^{'}$ is a vector of np, and *B* represents the index set $B=\{l:mod(l,n) \ne 0,l = 1,\cdots ,np\}$, does not include the n-th element. For simplicity, the paper will denote $\sum \limits _{l \in B}|\theta _{l}|$ by $|{\varvec{{\theta }}}_{B}|_{1}$. Therefore, by solving the lasso problem in (4) with respect to the parameter ${\varvec{{\theta }}}$, the solution of the SCC model (2) with lasso penalty can be obtained. The estimator of ${\varvec{{\beta }}}$ is given by $\hat{{\varvec{{\beta }}}}_{k}=\widetilde{{\textbf{H}}}^{- 1}\hat{{\varvec{{\theta }}}}_{k},k = 1,\cdots ,p$.

### Ethics declarations

This study does not involve human participants, and uses public data from the Public Health Science Data Center of Chinese Center for Disease Control and Prevention, so it was not approved by the Ethical Committee.

## Data Availability

Hand, Foot and Mouth Disease datasets generated during the current study are available in the Public Health Science Data Center of Chinese Center for Disease Control and Prevention (http://www.phsciencedata.cn/Share/index.jsp). The meteorological datasets are available in China Meteorological Data Network (http://data.cma.cn/).
